# Identifying Individual and Household Level Predictors of Undernutrition Among 6–59 Months Children in Bangladesh: A Multivariate Approach

**DOI:** 10.1002/puh2.70007

**Published:** 2024-10-30

**Authors:** Oumma Halima, Abira Nowar, Md. Hafizul Islam, Akibul Islam Chowdhury, Kazi Turjaun Akhter, Nazma Shaheen

**Affiliations:** ^1^ Institute of Nutrition and Food Science University of Dhaka Dhaka Bangladesh; ^2^ Department of Food Technology and Nutrition Science Noakhali Science and Technology University Noakhali Bangladesh; ^3^ Department of Nutrition and Food Engineering Daffodil International University Savar, Dhaka Bangladesh

**Keywords:** Bangladesh, child undernutrition, logistic regression, stunting, underweight, wasting

## Abstract

**Introduction:**

Globally malnutrition is considered one of the greatest threats to public health, particularly in low‐ and middle‐income countries. The present study examined the extent of undernutrition and the associated determinants among children aged 6–59 months in Bangladesh using data from the Nutrition Survey of Bangladesh (NSB), 2017–18.

**Methods:**

The sampling frame of NSB 2017–18 was specified by a 30 (locations) × 30 (households) cluster approach where 20 locations were from rural areas and the other 10 were from urban areas. Out of the sampled households, 566 children aged between 6 and 59 months were included in the analysis. Determinants of three anthropometric measures, weight‐for‐age (stunting), weight‐for‐height (wasting), and weight‐for‐age (underweight), were analyzed using a multiple logistic regression model.

**Result:**

The prevalence of stunting, underweight, and wasting of children was 34.5%, 40.6%, and 20.1%, respectively. Although the child's age, family size, cleanliness of the residential area, and food insecurity were significant determinants of malnutrition, inadequate and low‐quality protein consumption strongly predicted the development of underweight and stunting in infants and children. The odds of being stunted were 2 times (adjusted odds ratio [AOR]: 2.02, 95% confidence interval [CI]: 1.17–3.46; *p* = 0.011) and underweight was almost 2.5 times (AOR: 2.41; 95% CI: 1.27–4.56; *p* < 0.01) higher, respectively, among children who consumed inadequate amounts of protein.

**Conclusion:**

Because the percentage of children from wealthy families was relatively low in the present study, cleanliness of residential areas, food insecurity, and inadequate protein intake are likely to be key drivers of malnutrition in Bangladesh, which might be significantly reduced with better coverage of preventive nutrition programs.

## Graphical abstract

The study aimed to identify the determinants of undernutrition among children (6–59 months) using the Nutrition Survey of Bangladesh (NSB) data of 2017/18. Study findings revealed that higher family size, unhealthy living conditions, household food insecurity, and inadequate and low‐quality protein intake contributed to the different forms of undernutrition.

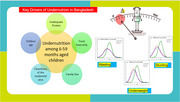

## 1 Introduction

Malnutrition is considered one of the crucial public health concerns around the world. The World Health Organization (WHO) declared it the single greatest threat to under‐five children, specifically those living in developing countries. Noticeably, undernutrition is more prevalent in South Asia. South Asia, which is famously known for the term “South Asian enigma,” contains 35% of the world's stunted and 15% of the world's under‐five wasted children along with micronutrient deficiencies [1]. About 50% of these undernourished children belong to three countries in South Asia, which are Bangladesh, India, and Pakistan [2]. The consequences of undernutrition may hamper severely both the physical and cognitive well‐being of an adult. Side by side, it inflates the odds of getting infections among under‐five children, which can result in increased morbidity and mortality [3]. It is estimated that these three countries can collectively comprise the highest level of disability‐adjusted life years (DALYs) due to child undernutrition [4]. The latest global estimates show an alarming figure that annually about half of the under‐five children's deaths can be attributed to undernutrition [5].

Bangladesh—the eighth most populous country in the world—has an under‐five mortality rate of 31 per 1000 live births [6]. Despite making tremendous progress in economic growth and health, the country is struggling to keep the prevalence of undernutrition indicators—stunting, wasting, and underweight—at a satisfactory level. The yearly reduction rate of stunting has become still, and it falls below the recommended global rate of 3.9% [7]. In addition, there has been no change in the prevalence of wasting, which remains at 22%, and the prevalence of wasting has increased from 8% to 11% [6]. Consequently, Bangladesh would lag in meeting the global nutrition targets for reducing stunting by the year 2025 [7] along with achieving its target levels committed in the country's Second National Plan of Action for Nutrition (NPAN2) [8]. To overcome the burden of undernutrition, the government of Bangladesh has adopted multi‐sectoral policies to accelerate the overall nutrition status of the population with a focus on the first 1000 days [8]. However, the after‐effects of the COVID‐19 pandemic [9] and the unaffordability of healthy diets [10] may make it more demanding for the country to tackle undernutrition and meet its targeted reduction rate. Thus, the country would need to continue the effective implementation of collaborative interventions to deal with undernutrition and fulfill the global benchmarks.

Malnutrition is interlinked with diverse factors. These factors can vary considerably not only across socio‐economic and demographic variables but also with time and location. The updated conceptual framework of 2020 by the United Nations Children's Fund (UNICEF) describes diet, care, food, practices, services, resources, norms, and governance under the categories of enabling, underlying, and immediate determinants of malnutrition [11]. Several studies around the world have been conducted to explore the risk factors for malnutrition among under‐five children, including Bangladesh. The existing literature on undernutrition and its factors in Bangladesh has identified various risk factors, such as region, gender, parental education level, wealth status, birth order and interval of children, home birth, and antenatal care visits, water, sanitation, and hygiene (WASH) practices, food insecurity, and low household dietary diversity as potential predictors of malnutrition among under‐five children for malnutrition [12, 13, 14, 15, 16, 17, 18, 19]. Despite available research on determinants of malnutrition, studies covering household and individual level variables as well as caregivers’ characteristics are sparse. To address this gap, a study involving a comprehensive set of potential variables is required, utilizing robust statistical modeling.

The study aims to identify the determinants of undernutrition among under‐five children using the Nutrition Survey of Bangladesh (NSB) data of 2017/18. The study would assess the prevalence of indicators of undernutrition and determine the contributing factors of undernutrition, including the child's characteristics as well as household‐level and caregiver's characteristics as the predictor variables. The study findings are expected to provide a better understanding of the determinants of malnutrition, which may assist in designing preventive nutritional programs focusing on the under‐five children of Bangladesh.

## 2 Methodology

### 2.1 Study Design and Data Source

The present study was designed to determine the predictors of undernutrition among children 6–59 months of age. This study conducted a secondary analysis of the NSB 2017–18, which is based on the first national NSB conducted in 1974–1975. The Institute of Nutrition and Food Science, University of Dhaka, conducted the NSB survey in 2017–18. This is the latest consumption survey in Bangladesh that included individual dietary intake data by weighing method. The NSB survey included participants from both rural and urban areas, which was representative of the entire Bangladesh.

### 2.2 Sample Selection and Inclusion Criteria

The NSB survey selected 30 sampling units following the probability proportion to size method to make a nationally represented sample of the population and a focus on the urban residents. Among these 30 sampling units, 20 were from rural areas, and the other 10 were from urban areas. Following a 30 × 30 sampling approach, 900 households were selected for data collection in the NSB survey. The survey was repeated in 10% of the households to ensure data validity, especially for dietary data by weighing method. From all the participants of the NSB survey, the present study included only the children 6–59 months of age and their caregivers (primarily mothers). A total of 591 children aged between 6 and 59 months from the NSB were included in the present study. After excluding incomplete data and implausible data (especially for nutritional status and *z*‐scores), 566 children, their caregivers, and household data were analyzed in the present study.

### 2.3 Data Collection by NSB Survey

In the NSB survey, a validated structured questionnaire (printed form) was used to collect data from the respondents (caregivers of the child) through face‐to‐face interviews [20]. The questionnaire covered information on the socio‐demographic characteristics of household members, household characteristics, economic conditions, food and non‐food costs, affordability, consumption data of the individual household member by 24‐h food weighing method, household food insecurity, and WASH conditions of residence. To ensure that the questionnaire was sufficient, appropriate, and clear‐speaking, it was pretested among 10% of the respondents. During these Muslim occasions and festival times, their lifestyle, including dietary patterns, substantially changes from the usual standpoint. Therefore, to avoid biases/errors in assessing usual dietary intake, the NSB survey did not collect any data during the month of Ramadan and special Muslim festivals. Moreover, the survey covered a full crop and weather cycle between April 2017 and March 2018. In the NSB survey, the dietary nutrient intake data (macronutrients, vitamins, and minerals) were obtained from the 24‐h dietary food intake using the food composition table for Bangladesh [21]. The detailed nutrient intake calculation procedure is given elsewhere [20].

Anthropometric parameters (height and weight) were taken for all children, aged 6–59 months, to assess their nutritional status. After receiving training, field personnel used the Length/Height Board of the UNICEF to measure a child's height or length. Children under 24 months were measured lying down with the infant's head resting against the infantometer's headboard (recumbent length). Height and length measurements were taken to the nearest centimeter. To obtain accurate readings, children were weighed using portable scales while wearing the barest minimum of clothing. Weights were recorded to the nearest 0.1 kg. Children who were unable to stand on the scale were weighed with the respondent, who was then weighed alone, and the difference was used to calculate the child's weight.

### 2.4 Study Variables

From the NSB survey, we used different outcome and predictor variables in the present study. In our study, we used the nutritional status (stunting, wasting, and underweight) of children 6–59 months as the outcome variables. Socio‐demographic characteristics, food insecurity, WASH, and dietary nutrient (macronutrients, vitamins, and minerals) intake data were used as predictor variables.

### 2.5 Outcome Variables

The outcome variables of the study were three forms of undernutrition (stunting, wasting, and underweight) among children of 6–59 months of age. To assess the nutritional status of children, WHO Anthro software (version 3.3.2) was used to calculate the height‐for‐age *z*‐score (HAZ), weight‐for‐age *z*‐score (WAZ), and weight‐for‐height *z*‐score (WHZ) from anthropometric data. WHO growth standard was adopted to classify children as stunted (HAZ < −2 SD), underweight (WAZ < −2 SD), and wasted (WHZ < −2 SD) [22]. Moreover, following WHO recommendations, any children with HAZ either above +6 or below −6 SD, WAZ above +5 or below −6 SD, and WHZ above +5 or below −5 SD were considered flag data for *z*‐score values and excluded from the analyses.

### 2.6 Predictor Variables

**FIGURE 1:**
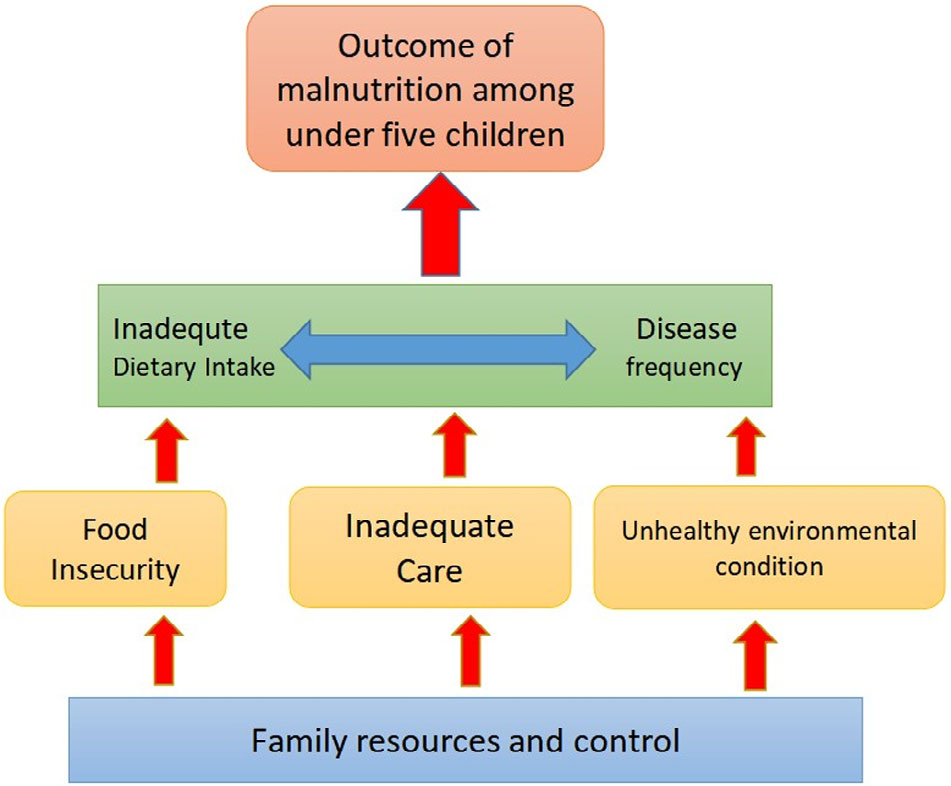
Conceptual framework of malnutrition by United Nations Children's Fund (UNICEF).

Following previous studies [12, 14–19] and the UNICEF conceptual framework of malnutrition among children [11], a number of variables at the individual and household levels were included in the analyses as potential predictors of undernutrition among children (Figure 1). The age of the children, their gender, energy and protein intake, and short‐term illness were considered a child's background variables. As caregivers’ background characteristics, age, gender, educational level, and employment status were included. Household characteristics, including household size, type of residence, hygiene and sanitation condition of residences, and food security status were also considered predictor variables.

Household food security condition was assessed using the Food Insecurity Experience Scale (FIES), which is an experience‐based metric for assessing the severity of food insecurity formulated by the United Nations Food and Agriculture Organization (FAO) [23]. The FIES questions are about the individual respondent's or the respondent's household's experiences. The questions are on self‐reported food‐related behaviors and experiences related to growing difficulty in acquiring food due to resource constraints that occurred in the previous year. Responses were recorded as either “yes” or “no” for each FIES question. For analytical purposes, “yes” responses were coded as 1, and “no” responses were coded as 0. Because the FIES consisted of eight questions, the total raw score ranged from 0 to 8, summing all the responses of all eight questions. The raw score defined individual food security status: food secure (score = 0), mild FI (score = 1–4; “yes” responses for the first four questions), moderate FI (score = 5–6; “yes” responses for fifth or sixth questions), and severe FI (score = 7–8; “yes” responses for seventh or eighth questions) [23].

Dietary intake of energy, protein, fat, vitamins, and minerals was also included in the present study. As a predictor variable of child nutrition, dietary intake adequacy of energy and protein were considered. We assessed the adequacy of protein and energy intake by evaluating the daily intake with ICMR‐recommended cut‐off values [24].

### 2.7 Statistical Analyses

Child's undernutrition (stunting, wasting, and underweight), and all the socio‐demographic characteristics at individual and household levels were summarized as frequencies and percentages. A chi‐square test was used to find the correlation between a child's undernutrition (stunting, wasting, and underweight) and other individual‐ and household‐level background characteristics. Further, logistic regression analyses were performed to determine the predictors of stunting, wasting, and underweight among children. WHO Anthro software (Version 3.3.2) was used for anthropometric data processing, and IBM Statistical Package for Social Science (SPSS), Version 23.0, was used to perform all statistical analyses. A *p* value less than 0.05 was considered to be statistically significant.

### 2.8 Regression Model Building

Three different multivariate logistic regression models were used to find the predictors of three different components of child undernutrition, stunting, wasting, and underweight as well as to adjust for potentially confounding effects. These outcome variables were binary: stunting [stunted, not stunted], wasting [wasted, not wasted], and underweight, not underweight. The underlying assumptions of the logistic regression models were examined before the final model building. Variables with significant associations at *p* values <0.25 in bivariate analyses were considered for inclusion in the regression models [25]. To evaluate whether a variable was statistically significant, *p* values of 0.05 were utilized. The stepwise forward entry method was used. The Pearson goodness of fit metric was used to evaluate how well the regression model fits the data. An adjusted odds ratio (AOR) with a 95% confidence interval (CI) was used to report the association.

## 3 Results

### 3.1 Characteristics of the Respondents

Table 1 represents the characteristics of the child, caregiver, and household. The percentage of children's sex was almost equally distributed. The majority of the children were over 1 year of age. In the case of caregivers (basically mothers), only 1 in 10 (9.7%) completed higher secondary/higher education. Most of the caregivers were unemployed (housewives). The condition of the residential area was moist and unhealthy in most households. It was shown that the majority of households (89.4%) did not purify their water before using it for cooking or drinking. Among the participants, 39.4% were from food‐secure households, whereas 43.6%, 10.2%, and 6.7% were from mild, moderate, and severe food‐insecure households, respectively.

**TABLE 1:** Demographic characteristics of the respondents and their household.

Child		Caregiver		Household	
Characteristics	*n* (%)	Characteristics	*n* (%)	Characteristics	*n* (%)
Total	566		566		566
**Sex** Male Female	284 (50.2) 282 (49.8)	**Age (years)** <35 35–55 >55	514 (90.8) 48 (8.5) 4 (0.7)	**Household size** ≤5 persons >5 persons	307 (54.2) 259 (45.8)
**Age (months)** 6–12 13–36 3759	65 (11.5) 285 (50.4) 216 (38.2)	**Education** No formal education Primary Secondary Higher secondary University/Above	75 (13.3) 204 (36.0) 232 (41.0) 33 (5.8) 22 (3.9)	**Surrounding environment around living room** Dry Clean Damp Dirty	80 (14.1) 312 (55.1) 50 (8.8) 124 (21.9)
**Short‐term illness** ^ǂ^ Yes No	178 (31.4) 388 (68.6)	**Occupation** Unemployed Self‐employed Service (govt/private)	550 (97.2) 3 (0.5) 13 (2.3)	**Wall and floor of living room** Dry Clean Damp Dirty	24 (4.2) 137 (24.2) 259 (45.8) 146 (25.8)
				**Method of water purification** Boiling Filtration Tablet Not purified	35 (6.2) 3 (0.5) 22 (3.9) 506 (89.4)
				**Area of residence** Rural Urban	284 (50.2) 282 (49.8)
				**Household food insecurity** Food secure Mild food insecure Moderate food insecure Severe food insecure	223 (39.4) 247 (43.6) 58 (10.2) 38 (6.7)

^ǂ^ Short‐term illness indicates the last 2 weeks’ morbidity status of children reported by the respondents.

### 3.2 Dietary Intake

**FIGURE 2:**
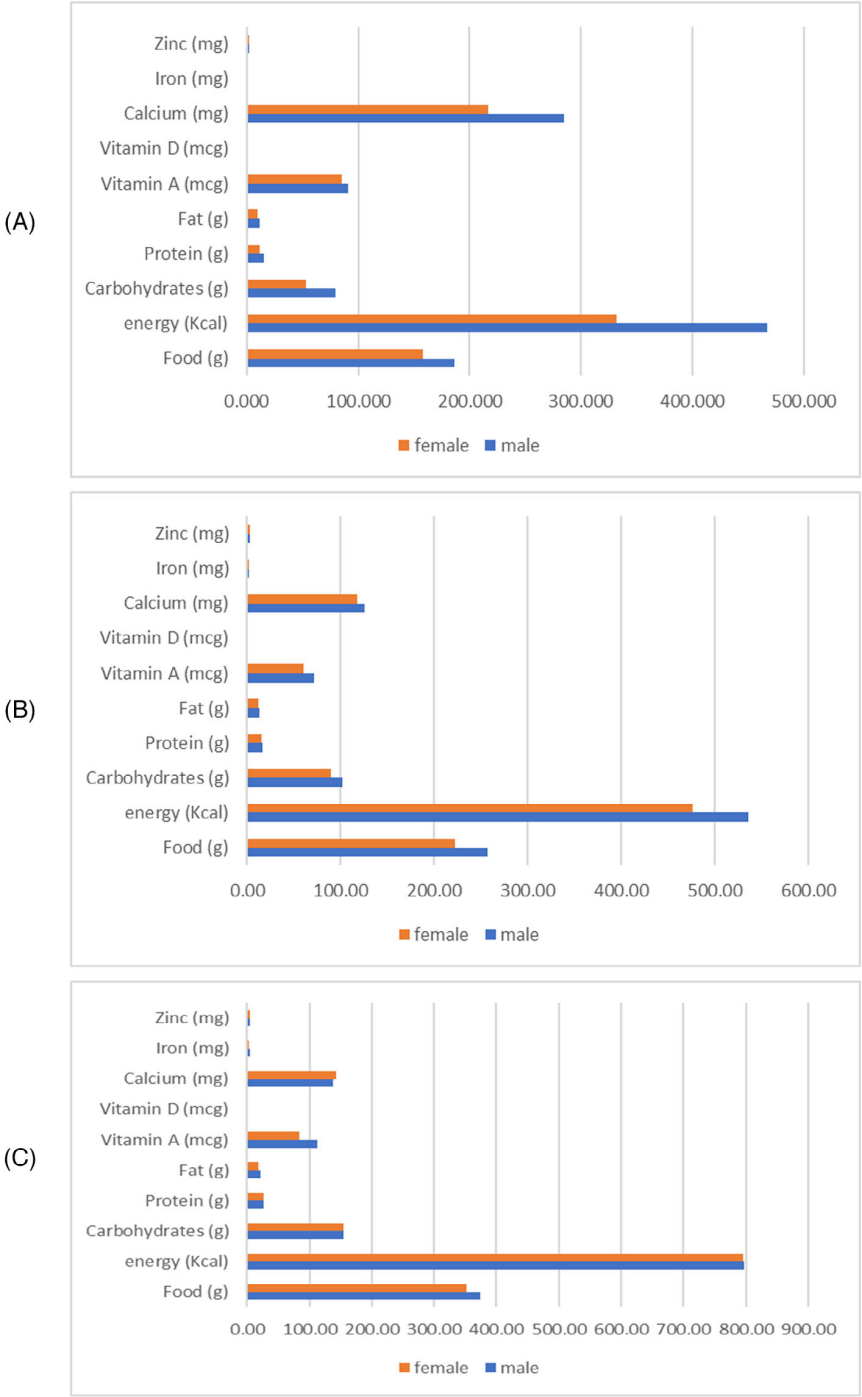
Average nutrient intake of children aged: (A) 6–12 months; (B) 13–36 months; (C) 37–59 months.

The average intake of different nutrients by different age and sex groups is depicted in Figure 2. Overall, the mean intake of public health significance nutrients was higher among the male children compared to the female. The mean consumption of carbohydrates, protein, and fat was about 114.9 g (range: 2.34–395.4 g), 19.7 g (range: 0.24–78.2 g), and 15.14 g (range: 0.11–125.04 g), respectively. While estimating the consumption of micronutrients, the mean consumption of vitamin A, D, retinol, and beta‐carotene was about 80.8, 0.44, 40.8, and 424.57 mcg, respectively.

### 3.3 Malnutrition and Covariates

**FIGURE 3:**
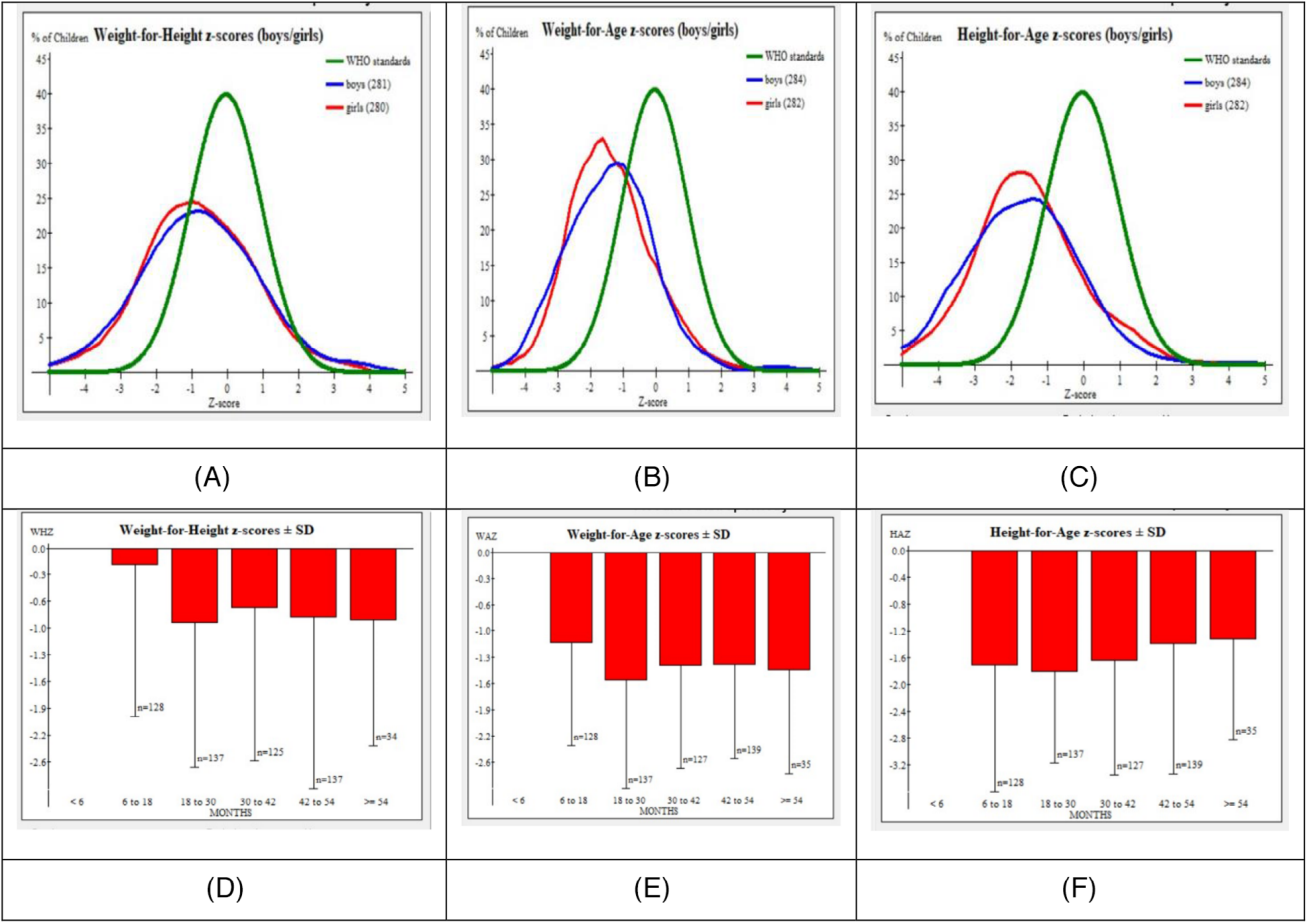
Prevalence of wasting, underweight, and stunting by sex and age group; (A) prevalence of wasting among 6–59 months aged children by sex group; (B) prevalence of underweight among 6–59 months aged children by sex group; (C) prevalence of stunting among 6–59 months aged children by sex group; (D) distribution of wasting by age group; (E) distribution of underweight by age group; (F) distribution of stunting by age group.

This study sought to assess the prevalence and risk factors of undernutrition among children aged 6–59 months in Bangladesh. Results showed the prevalence of stunting, underweight, and wasting of 34.5%, 40.6%, and 20.1%, respectively, with severe cases of 12.7%, 9.4%, and 6% being observed in each category. The prevalence of wasting, being underweight, and stunting by different sexes and age groups has been shown in Figure 3.

The distribution of malnourished children according to the demographic profile and other covariates is presented in Table 2. A significant association was observed among malnourished children (stunted, wasted, and underweight) with their age and short‐term illness history (*p* < 0.05). Caregivers’ educational background also showed a significant association with stunting and underweight children. The household characteristics, such as household size, hygiene of residence, and food insecurity status, also showed a significant relation with stunting, wasting, and underweight children (*p* < 0.05). The surrounding environment of the household's living room showed a statistically significant correlation with the wasting status of children.

**TABLE 2:** Distribution of stunted, wasted, and underweight children according to children, caregiver, and household characteristics.

		Stunting		Wasting		Underweight	
Characteristics	*N*	%	*p* value	%	*p* value	%	*p* value
**Sex of the child**							
Male	284	36.9	0.362	19.4	0.660	40.5	0.945
Female	282	32.6		20.9		40.8	
**Age of the child (months)**							
6–12	65	52.3	0.002	7.7	0.023	29.2	0.036
13–36	285	35.1		22.8		45.3	
37–59	216	28.2		20.5		38.0	
**Short‐term illness of the childǂ**							
Yes	178	44.4	0.001	25.8	0.023	48.9	0.007
No	388	29.9		17.6		36.9	
**Age of the caregiver (years)**							
<35	514	33.7	0.343	19.1	0.030	39.3	0.124
35–55	48	43.8		34.0		54.2	
>55	4	25.0		0.0		50.0	
**Education of the caregiver**							
No formal education	75	40.0	0.012	21.6	0.392	41.3	0.002
Primary	204	42.2		21.6		50.5	
Secondary	232	28.4		19.4		34.5	
Higher secondary	33	24.2		24.2		36.4	
University/Above	22	22.7		4.5		18.2	
**Occupation of the caregiver**							
Unemployed	550	34.7	0.433	19.7	0.211	40.4	0.599
Self‐employed	3	0.0		33.3		33.3	
Service (govt/private)	13	30.8		38.5		53.8	
**Household size**							
≤5 persons	307	10.4	<0.001	12.1	<0.001	17.3	<0.001
>5 persons	259	62.9		29.7		68.3	
**Surrounding environment of the living room**							
Dry	80	40.0	0.094	3.8	<0.001	35.0	<0.001
Clean	312	34.0		1.3		21.2	
Damp	50	20.0		6.0		46.0	
Dirty	124	37.9		84.6		91.1	
**Wall and floor of living room**							
Dry	24	41.7	<0.001	8.3	<0.001	33.3	<0.001
Clean	137	26.3		0.7		5.1	
Damp	259	29.7		4.3		29.0	
Dirty	146	49.3		68.5		95.9	
**Method of water purification**							
Boiling	35	20.0	0.078	31.4	0.286	40.0	0.413
Filtration	3	0.0		0.0		0.0	
Tablet	22	22.7		22.7		31.8	
Not purified	506	36.2		19.4		41.3	
**Area of Residence**							
Rural	284	35.2	0.385	22.9	0.066	45.8	0.012
Urban	282	33.7		17.4		35.8	
**Household food insecurity**							
Food secure	223	22.4	<0.001	17.9	<0.001	35.9	<0.001
Mildly insecure	247	34.0		16.2		38.1	
Moderate insecure	58	50.0		14.0		43.1	
Severe insecure	38	84.2		68.4		81.6	

### 3.4 Associated Factors of Undernutrition

Table 3 demonstrates the factors affecting the nutritional status of children. The odds of being stunted children were 2.76 times higher among children aged 6–12 months compared with children aged 37–59 months (AOR: 2.76, 95% CI: 1.21, 6.28; *p* = 0.015). The odds of being stunted and underweight were significantly higher among children from households with more than five members (*p* < 0.05). Compared with a dry environment, it was found that the children in an unhealthy environment were more likely to be wasted (AOR: 47.3, 95% CI: 11.52, 194.46; *p* < 0.001). The cleanliness of residential areas also showed a significant association with the likeliness of underweight children. The children from mild, moderate, and severe food‐insecure households were more likely to be stunted compared to the children from food‐secure households. The regression model showed a significant association of protein intake by children with stunting and underweight. The children who consumed inadequate protein were more likely to be stunted (AOR: 2.02, 95% CI: 1.17–3.46; *p* = 0.011) and underweight (AOR: 2.41; 95% CI: 1.27–4.56; *p* < 0.01) compared to the children who consumed an adequate amount of protein.

**TABLE 3:** Associated risk factors of stunting, wasting, and underweight among children 6–59 months.

Characteristics	Stunting		Wasting		Underweight	
	AOR (95% CI)	*p* value	AOR (95% CI)	*p* value	AOR (95% CI)	*p* value
**Age of the children (months)**						
6–12	2.76 (1.21–6.28)	0.015	0.35 (0.06–2.03)	0.934	0.44 (0.17–1.16)	0.095
13–36	1.57 (0.93–2.69)	0.093	0.82 (0.33–2.08)	0.353	1.26 (0.66–2.41)	0.477
37–59	1		1		1	
**Short‐term illness** ^ǂ^						
Yes	0.73 (0.43–1.24)	0.244	0.58 (0.20–1.69)	0.321	0.49 (0.26–0.92)	0.027
No	1		1		1	
**Age of the caregiver (years)**						
<35	1	0.441	1	0.886	1	0.176
35–55	1.39 (0.59–3.25)	0.623	0.89 (0.18–4.24)	0.999	2.14 (0.71–6.41)	0.981
>55	1.87 (0.15–22.81)		0.001 (0.00–0.00)		1.04 (0.06–17.37)	
**Education of the caregiver**						
No formal education	1.03 (0.25–4.34)	0.922	—	—	1.20 (0.18–7.68)	0.846
Primary	0.82 (0.21–3.17)	0.817			1.87 (0.31–11.21)	0.493
Secondary	0.75 (0.19–2.90)	0.712			0.54 (0.08–3.23)	0.497
Higher secondary	0.63 (0.12–3.44)	0.654			0.53 (0.06–4.42)	0.559
University/Above	1				1	
**Household size**						
≤5 persons	1	<0.001	1	0.609	1	<0.001
>5 persons	24.5 (13.7–43.9)		1.28 (0.49–3.31)		15.15 (7.74–29.68)	
**Surrounding environment around living room**						
Dry	1	0.277	1	0.267	1	0.042
Clean	0.69 (0.33–1.41)	<0.001	0.39 (0.07–2.03)	0.624	0.41 (0.17–0.97)	0.153
Damp	0.14 (0.05–0.43)	<0.001	1.58 (0.25–10.05)	<0.001	2.32 (0.73–7.31)	0.196
Dirty	0.09 (0.04–0.25)		47.33 (11.52–194.46)		2.16 (0.67–7.00)	
**Wall and floor of living room**						
Dry	1	0.671	1	0.239	1	<0.001
Clean	1.46 (0.41–5.17)	0.474	0.17 (0.09–3.20)	0.310	0.09 (0.02–0.34)	0.422
Damp	0.75 (0.23–2.48)	0.252	0.32 (0.04–2.91)	0.303	0.63 (0.21–1.93)	<0.001
Dirty	2.63 (0.68–10.14)		3.09 (0.36–26.59)		29.07 (6.47–130.65)	
**Household food insecurity**						
Food secure	1	0.034	1	0.571	1	0.940
Mildly insecure	1.86 (1.08–3.22)	<0.001	1.33 (0.49–3.58)	0.612	1.03 (0.52–2.05)	0.414
Moderate insecure	3.03 (1.31–6.97)	0.039	0.67 (0.15–3.11)	0.342	0.65 (0.23–1.82)	0.678
Severe insecure	34.53 (9.84–121.15)		2.57 (0.37–18.01)		0.73 (0.16–3.25)	
**Energy intake**						
Adequate	1	0.153	1	0.493	1	0.166
Inadequate	0.39 (0.10–1.43)		2.71 (0.16–46.96)		3.45 (0.59–19.90)	
**Protein intake**						
Adequate	1	0.011	1	0.951	1	0.007
Inadequate	2.02 (1.17–3.46)		0.97 (0.38–2.45)		2.41 (1.27–4.56)	

^ǂ^ Short‐term illness indicates the last 2 weeks' morbidity status of children reported by the respondent.

## 4 Discussion

In the present study, the data from the NSB undertaken from 2017 to 2018 was analyzed to investigate the prevalence and risk factors of undernutrition among children aged 6–59 months in Bangladesh. The prevalence of stunting, underweight, and wasting of children was 34.5%, 40.6%, and 20.1%, respectively. The malnutrition prevalence among under‐five children in our study was higher when compared to the national prevalence obtained from the 2017–18 BDHS report [26], which could be because of different age groups, time frames, and sampling techniques. Though Bangladesh has made considerable progress in increasing national‐level food availability and individual‐level energy intake, other essential nutrients are still far below the nutrient requirements. The possible reason for this high prevalence of malnutrition partly might be the imbalance in macro‐ and micro‐nutrient intake, hygiene and sanitation condition of residence, frequency of illness, household size, and education level of the caregiver.

Our study found stunting rates higher among the 6–12 month age group, which is consistent with the results obtained from a community‐based cross‐sectional survey in India [27], a hospital‐based survey in Nigeria [28], and an analytical observational study in Central Lombok [29]. This can be better explained by minimum dietary diversity, minimum meal frequency, and minimum acceptable diet. From the present study findings, only 26.6% of children aged 6–23 months were fed a minimum acceptable diet. Less than 50% (44.4%) received a minimum of five food groups out of eight, and 50% received their age‐appropriate minimum number of meals. Feeding according to IYCF recommendations increases with a child's age. Adherence to the Infant and Young Children Feeding (IYCF) practices was better in children aged 18–23 months.

The significant association of malnutrition indicators with family size observed herein was coherent with the studies conducted in India [30], Uganda [31], and Ethiopia [32, 33]. A household's responsibility to feed all of its members and their children with the balanced nutritious food increases with the number of family members living there. Furthermore, the higher the number of children in households, the more unlikely it is that every child gets proper care and time, which increases the likelihood that they may suffer from malnutrition.

Children residing in unhealthy places were more likely to be underweight as compared to their peers residing in dry or clean places. About 96% of children living in unhealthy rooms were reported to be underweight, which could be the occurrence of frequent short‐term illnesses, for example, diarrhea, dysentery, pneumonia, measles, tuberculosis, anemia, fever, and cough. The association between household sanitation and malnutrition has been established by research findings from Pakistan Demographic and Health Survey (PDHS) 2017–2018 [34]. This statement is further strengthened by the UNICEF conceptual framework, which describes insufficient access to clean WASH as an underlying contributing factor to undernutrition [11].

Malnutrition is the most severe consequence of food insecurity. Food insecurity was found to be associated with higher odds of being stunted among children in the current study. Several studies have found a similar association [35, 36]. Likewise, there was a statistically significant correlation between household food insecurity and stunting among children in Malaysia and Pakistan [37, 38].

In this study, inadequate and low‐quality protein consumption predicted the development of underweight and stunting in infants and children, which was supported by several observational studies. A considerable correlation has been shown between higher protein consumption and a decrease in both stunting and wasting in the studies that looked at the relationship between protein intake and childhood malnutrition, particularly linear growth [39, 40]. For instance, after adjusting for pertinent confounders, a cross‐sectional study involving 3150 infants and toddlers from the Democratic Republic of the Congo, Zambia, Guatemala, and Pakistan revealed that eating adequate meat and fish was linked to a lower risk of both stunting and wasting [41]. Besides, several intervention studies conducted in Africa were in line with the findings of these observational studies [42, 43]. Stunting in the context of protein and amino acids, which, beyond supplying essential amino acids for protein synthesis involving linear growth, can be further explained by the quality, not just by the quantity. A recent study conducted in India found the association of protein quality as assessed by DIAAS with the prevalence of stunting [44].

When evaluating the findings of this study, a few limitations should be taken into account. Because malnutrition is a long‐term cumulative process, the direction of the associations observed in this cross‐sectional study cannot be ascertained. Therefore, it is not possible to draw concrete conclusions about the causal impact of the key indicators listed here and prioritize any particular interventions without conducting a cost–benefit analysis. Moreover, a considerable portion of the data obtained was based on self‐reported information provided by the caregivers. As a result, there could be a tendency for caregivers to under‐report or over‐report. Furthermore, recall bias could equally come into play as caregivers may not properly recall all required information. Another drawback was that children from low‐ and middle‐income families made up the majority of the study's participants. The percentage of children from wealthy families was relatively low. However, we analyzed the data using reliable statistical methods, and we are still confident in the findings and information provided here.

As strengths, this research accounted for major risk factors and confounders of undernutrition in the analysis. Moreover, the study used the nationally representative nutrition survey data, which makes it possible to identify the immediate, underlying, and basic causes of malnutrition status in infants and children. In addition, a 24‐h food weighing method for measuring household and individual consumption was used with information on the dishes and ingredients used and consumed, followed by details on the quantity as well as quality, and food taken outside the home was recorded using the recall method, which are rare components in other studies. Therefore, this study analysis will help in the better understanding of the burden of undernutrition and its associated risk factors among under‐five children of the study population in Bangladesh.

## 5 Conclusion

The study highlighted the prevalence and risk factors of undernutrition among children aged 6–59 months in Bangladesh. A higher prevalence of stunting, wasting, and underweight was found among these children. Higher family size, unhealthy living conditions, household food insecurity, and inadequate and low‐quality protein intake contributed to the different forms of undernutrition among the children. However, to develop a strategy to eliminate child undernutrition in Bangladesh in a long‐term, sustainable way, it is important to ensure the involvement of the government, non‐governmental organizations, and community and working together. Therefore, with greater coverage of nutrition‐specific (e.g., breastfeeding promotion, balanced energy and protein, and multiple micronutrient supplementation) and nutrition‐sensitive (e.g., improving socio‐economic status, improving access to water and sanitation facilities), the burden of malnutrition may be significantly reduced.

## Author Contributions

**Oumma Halima**: data curation, formal analysis, writing–original draft, methodology, software. **Abira Nowar**: writing–original draft. **Md. Hafizul Islam**: writing–original draft. **Akibul Islam Chowdhury**: writing–original draft. **Kazi Turjaun Akhter**: writing–review and editing. **Nazma Shaheen**: conceptualization, investigation, funding acquisition, validation, visualization, writing–review and editing, project administration, supervision, resources.

## Ethics Statement

Because the current study had been conducted from not individually identifiable secondary data, IRB review was not required for this study.

## Conflicts of Interest

The authors declare no conflicts of interest.

## Transparency Statement

The corresponding author (Dr. Nazma Shaheen) affirms that “the manuscript is an honest, accurate, and transparent account of the study being reported; that no important aspects of the study have been omitted; and that any discrepancies from the study as planned (and, if relevant, registered) have been explained”.

## Data Availability

The original dataset is available in the FAO/WHO GIFT platform (Link: https://www.fao.org/gift‐individual‐food‐consumption/data/en). The datasets generated during this study are available from the corresponding author on a reasonable request.
